# Age-specific responses to spring temperature in a migratory songbird: older females attempt more broods in warmer springs

**DOI:** 10.1002/ece3.673

**Published:** 2013-08-13

**Authors:** L Bulluck, S Huber, C Viverette, C Blem

**Affiliations:** 1 Department of Biology, Virginia Commonwealth UniversityRichmond, Virginia; 2 Department of Fisheries Science, Virginia Institute of Marine SciencesGloucester Point, Virginia; 3 Center for Environmental Studies, Virginia Commonwealth UniversityRichmond, Virginia

**Keywords:** age-specific reproduction, multibrooding, neotropical migratory bird, nest initiation date, phenology, prothonotary warbler, *Protonotaria citrea*, resource peaks

## Abstract

Increasing global temperature has led to an interest in plasticity in the timing of annual events; however, little is known about the demographic consequences of changing phenology. Annual reproductive success varies significantly among individuals within a population, and some of that variation has to do with the number of broods attempted by reproducing adults. In birds, female age and the timing of reproduction are often predictors of multiple breeding. We hypothesize that double brooding rates may be affected by spring temperature and that the response may vary with female age. We used a long-term reproductive data set for a migratory songbird, the prothonotary warbler (*Protonotaria citrea*) to assess which factors influence (a) an individual female's probability of double brooding and (b) the annual variation in population-level double brooding rates. We found that older and earlier nesting birds are more likely to double brood, and that there is no evidence for senescence with regard to this trait such that the oldest females were most likely to double brood. Previous experience with double brooding (i.e., whether the female double brooded in the previous year) significantly increased the probability of doing so again. When assessing annual variation in the double brooding rate, we found an interaction between spring temperature and the proportion of older females in the population. Specifically, older females are more likely to double brood in years with warmer springs, but this relationship was not seen for younger females. Previous studies have shown that warmer temperatures lead to earlier and narrower peaks in resources and we hypothesize that these peaks are more available to older and earlier arriving females, enabling them to successfully raise more than one brood in a season. Understanding how different age classes respond to changing environmental conditions will be imperative to managing declining species.

## Introduction

Increasing global temperature has led to an interest in the plasticity of the timing of annual events; however, little is known about the demographic causes or consequences of changing phenology (Forest and Miller-Rushing 2010). Generally, studies on birds suggest that warming temperatures have led to a mismatch between the timing of reproduction and resources needed for reproduction (Visser et al. 1998; Both et al. 2006); however, studies that consider potential age-specific responses to climate warming are rare (Knudson et al. 2011). Indeed, scientists have recently called for an increased consideration of life history when assessing variation in phenology in an effort to better understand the demographic effects of our changing global climate (Forest and Miller-Rushing 2010).

Annual reproductive success is an important demographic contributor to population growth rates, and reproductive output varies significantly among individuals within a population. The number of offspring produced in a given year is especially important for short-lived species; these species are more likely to invest in the current year's reproductive attempt, even at the risk of their own survival (Stearns 1976; Newton 1989). An individual can increase their reproductive success by either increasing the number of offspring produced per breeding attempt or by increasing the number of breeding attempts per season. Many bird species lay one or more clutches of eggs after successfully rearing a first brood and such multiple brooding has been shown to significantly increase an individual's reproductive output (Saether and Bakke 2000) and explain ∼20% of the variation in annual fecundity (Nagy and Holmes 2004). However, there are costs associated with raising multiple broods, particularly with regard to lower survival and recruitment of young produced from these later breeding attempts (Morrison 1998; Tarof et al. 2011; Serra et al. 2012). Such costs may be greater for younger breeders, especially when environmental conditions are poor (Aroyyo et al. 2007).

Despite these costs, many species of bird are able to raise more than one brood of young in a year and tend to have higher site fidelity when they have been able to do so (Hoover 2003). Not all individuals in a population will multibrood and there is interest in understanding what variables contribute to whether or not a female will attempt it. Previous studies suggest that older females (Kluyver 1951; Geupel and DeSante 1990; but see Ogden and Stutchbury 1996; Nagy and Holmes 2005a) who initiate egg production earlier (Verboven and Verhulst 1996; Morrison 1998; Møller 2008; Monroe et al. 2008) and nest in higher quality habitats (Perrins 1965; Newton 1998) or territories (Rodenhouse and Holmes 1992; Nagy and Holmes 2004, 2005a,b) are more likely to contribute to a population's growth. Furthermore, because food resources are often seasonally pulsed, the timing of reproduction relative to peaks in food abundance has also been demonstrated to affect double brooding rates (Verboven et al. 2001; Husby et al. 2009).

Although prior studies have provided much needed information on what influences individual reproductive success, they primarily use data from only a few breeding seasons and individuals. Long-term data sets are required to understand factors that are driving annual variation and to accumulate enough demographic data to understand how changing environmental conditions limit population growth. An invaluable asset of long-term datasets is the ability to obtain a population of known-aged individuals whereby age effects can be clearly understood. Such long-term studies exist, but few assess multiple brooding explicitly; exceptions include a study of wood thrush (*Hylocichla mustelina*) where no environmental factors were assessed (Brown and Roth 2009) and great tits (*Parus major*, Visser et al. 2003; Husby et al. 2009). Double brooding rates in great tit populations have generally declined over time and with increasing temperature, likely due to a mismatch in the timing of reproduction and the pulse of food resources (Husby et al. 2009; Reed et al. 2013).

To address the issue of increasing spring temperatures as it pertains specifically to double brooding in a Neararctic Neotropical migratory bird, we use a long-term (17-year) data set of prothonotary warbler (*Protonotaria citrea*) reproduction in artificial nest boxes along the lower James River in eastern Virginia, USA. Such long-term data sets provide insight into the plasticity in annual reproductive events and are comprised predominantly of known-aged individuals compared with shorter demographic studies. Our objectives were first to address the factors that influence an individual female's probability of double brooding and then to assess what factors are associated with the interannual variation in double brooding rates (i.e., the proportion of females that double brood) in this population.

## Methods

### Species and study sites

**Figure 1:**
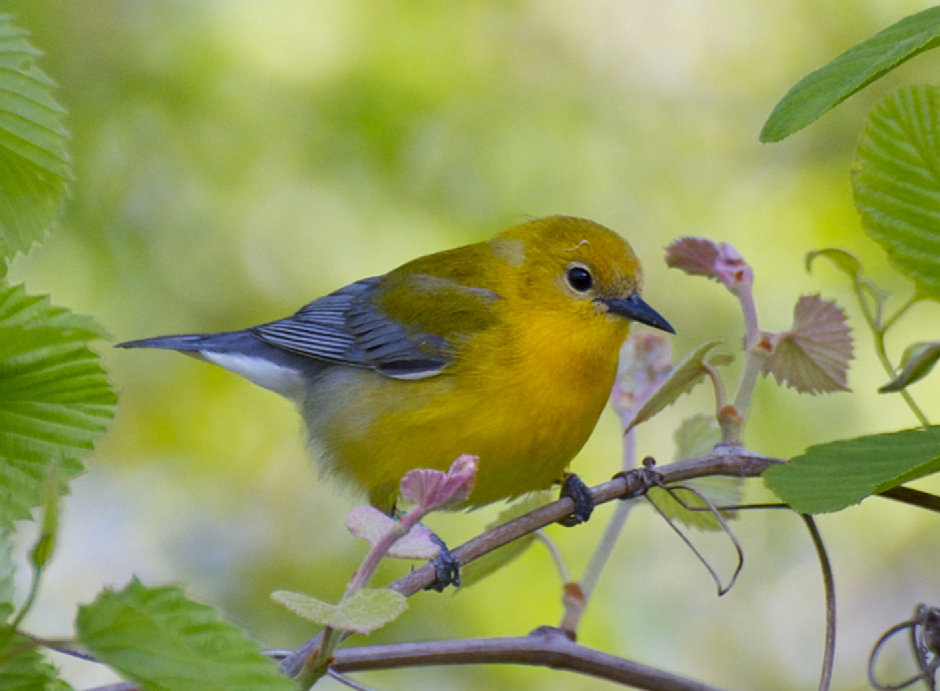
Female Prothonotary Warbler (*Protonotaria citrea*) in Henrico County, VA April 2013. Photo by John Wojcikiewicz.

The prothonotary warbler (Fig. 1) is a Neotropical migratory songbird that inhabits bottomland hardwood forests and cypress swamps throughout the eastern United States. It is one of only two cavity-nesting warblers in North America, and will readily nest in man-made boxes (Petit 1999). Clutch size is 3–7 eggs with one egg being laid each day and incubation beginning on the last day an egg is laid. The 12- to 14-day incubation period is carried out solely by the female and the nestling period is 9–10 days where both males and females feed the nestlings. Once fledged, both parents continue to feed the fledglings, however, if the female initiates a second brood, the male takes primary responsibility of feeding (Petit 1999).

A long-term study of prothonotary warbler breeding biology in the lower James River, Virginia was initiated by Charles and Leann Blem in 1987 (Blem and Blem 1991). The number of nest boxes in that study system has increased from 141 boxes at Presquile National Wildlife Refuge (NWR) in 1987 to ∼650 boxes across five sites in 2012 (Presquile National Wildlife Refuge, Dutch Gap Conservation Area, Deep Bottom Park in Henrico County, Virginia Commonwealth University Rice Center, and White Bank). This study focused on sampling efforts at Presquile NWR and Deep Bottom Park from 1995 to 2012 when sampling was consistent in the latter part of the season when second clutches are laid. All boxes are mounted on metal conduit poles over water and near the shore (<5 m at high tide) and the density of birds did not vary significantly from year to year as nearly all boxes are typically occupied in a season. At low tide, some boxes are over mud/rock and therefore exposed to terrestrial predators. In general, predation is very low (<10–15%) compared to natural cavities (27–43% [Walkinshaw 1953; Petit 1989; Flaspohler 1996; Hoover 2006]) and often occurs when snakes are able to access the boxes from low, overhanging vegetation. Flooding of boxes at high tide and with increased rain is also a cause of nest failure in both natural cavities and nest boxes (Petit 1989).

### Field methods

Nest boxes were checked two to three times per week during the breeding season, and nest contents were recorded as well as signs of fledging (feces in and around a flattened nest cup). Incubating females were captured at the boxes and were banded with a metal USFWS band (Permit #22751) and aged as second year or after second year according to feather criteria in Pyle (1997). Nestlings were also banded on days 4–8 of the nestling period. We were able to determine actual ages of recaptured individuals that were banded as nestlings or often during their first year of breeding based on plumage. Actual ages were not known for some individuals (<20%); adults of unknown age at banding (after-hatch year) and those known to be at least in their second year of breeding (after second year). The former were given an age of 1.5 and the latter as 2.5 (i.e., a bird was *at least* 2 years old) and for some comparisons, these ages were rounded up to the next year. Captured females ranged in age from 1 (first year breeding) to 9 (ninth year breeding). For more information regarding the details of field data collection see Blem and Blem (1991).

### Statistical methods

#### Probability of individual female double brooding

Any nest initiated (first egg laid) on or before June 1 (Julian date 152) was considered an early clutch and used in the analysis; 80% of all first clutches were initiated before June 1 and successful nests initiated after this date were never followed by a second brood. Therefore, excluding first nest attempts after June 1 is conservative as these individuals would bias our results with regard to the effect of clutch initiation date on double brooding. Predation is rare in our system, though renesting was common after predation when it did happen. However, we were interested in double brooding only and so excluded these from analysis. To determine which variables influenced the probability of an individual female double brooding, we performed a generalized linear mixed model (GLMM, binomial family and logit link), with probability of double brooding as the response variable and female age, clutch initiation date for the first nesting attempt of the season, and clutch size for the first clutch as predictors. Year and female band number were included as random effects to account for repeated measures of females over time. We expected older females to double brood more than younger females, earlier breeders to double brood more than later breeders, and females with smaller first clutches to double brood more than those with larger first clutches. We were also interested in whether or not breeding effort in the previous year influenced a female's decision to double brood so for a subset of individuals for which we had data for consecutive years we ran the same GLMM and added a variable for whether or not the bird double brooded in the previous year (DB_(YRn-1)_). We were not able to include body condition in this analysis because tarsus and mass have only recently (since 2009) been consistently measured for all females. In recent years (2009–2012, *N* = 280 females), however, body condition calculated as the residuals from a regression of tarsus and mass does not correlate with age (*P* = 0.166), nest initiation date (*P* = 0.744), or the number of eggs laid in the first clutch (*P* = 0.337).

#### Proportion of females double brooding each year

We expected that there would be significant interannual variation in double brooding rates, and were therefore interested in factors that contributed to interannual variation across the 17 years of the study. Using the proportion of females that double brood in a given year as the response variable, we performed a multiple linear regression with mean daily minimum temperature in April, mean clutch initiation date, and the proportion of older females in the population (≥3rd year breeding). We used mean daily minimum April temperature in each year to represent limitations imposed by spring temperature because warbler's primary food at this time of their annual cycle is caterpillars whose emergence is temperature dependent and are specifically limited by minimum daily temperatures (Visser et al. 2006; Valtonen et al. 2011). Furthermore, April is when the prothonotary warblers arrive and begin laying their first clutch in our sites. Analyses were also run with April mean temperature and the results were very similar. We obtained April temperature data from Weather Underground for Richmond, VA (wunderground.com). We included the proportion of older females and mean clutch initiation date because we found both of these to influence the probability that a female will double brood (see results). We also included interactions between the proportion of older females and mean minimum April temperature as well as between the proportion of older females and mean clutch initiation date. These interactions address our hypotheses that spring temperature (an indirect measure of caterpillar emergence) could influence younger and older birds differently as they arrive and initiate nests at different times (Blem et al. 1999), and therefore differ in their ability to initiate clutches.

All statistical analyses were conducted in the R statistical package (version 2.15.1; Vienna, Austria). We assessed colinearity among predictors and normality before running the analysis and found pairwise correlations (*r* values) to be <0.5 for the individual female analysis (*N* = 2027) and <0.6 for the annual analysis (*N* = 17) and found no significant deviance from normality.

## Results

### Probability of individual female double brooding

**Figure 2:**
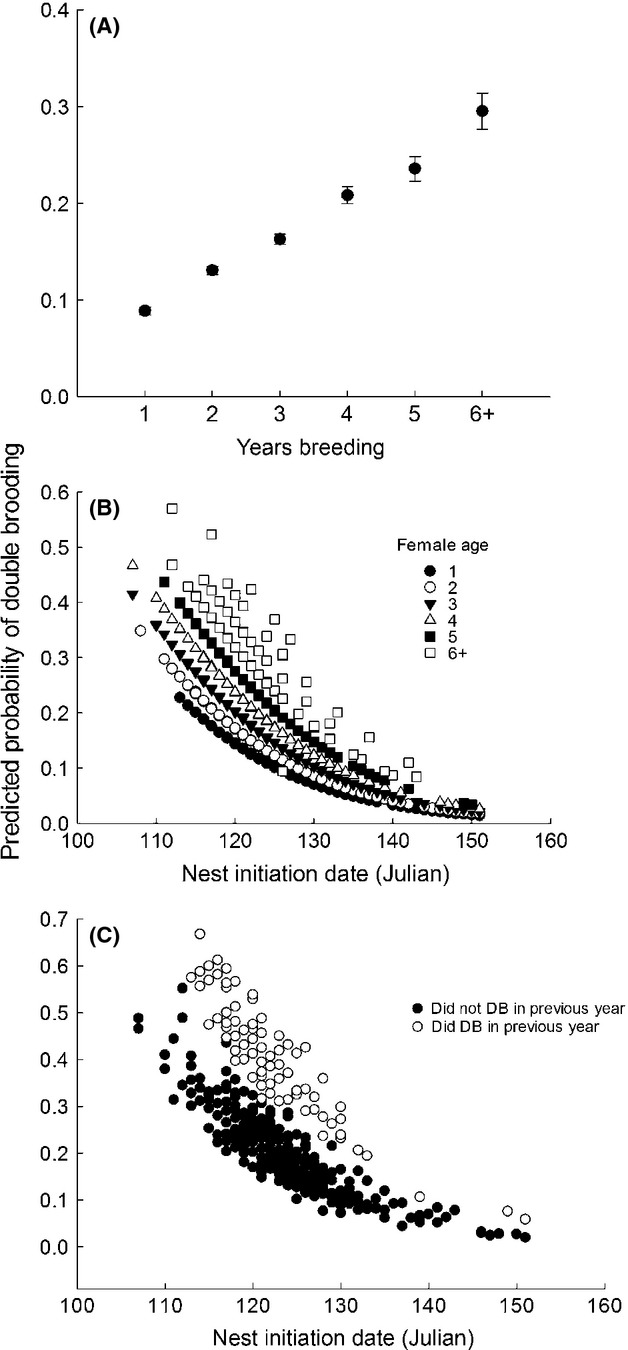
The predicted probability of double brooding in prothonotary warblers increases with female age (A) and for birds who initiate their nests earlier (B) based on output from the GLMM including all individuals. The predicted probability of double brooding also increases for birds that double brood in the previous year (C) based on output from the GLMM including only the individuals for which we have consecutive years of breeding data.

Across all breeding events for early breeders initiating their first nests on or before June 1 (*N* = 2027 from 1305 different females) between 1995 and 2012, 15.79% of those with successful first clutches went on to lay a second clutch of eggs (*N* = 320). If late breeders were included (*N* = an additional 385 breeding events), 13.3% of all breeding females across all years produced a second clutch after successfully rearing a first brood. On average, females that double brooded fledged 3.0 more young per year (6.8 compared with 3.8, *P* < 0.0001) than females that did not double brood, which nearly doubled their annual fecundity. The overall GLM model was significant (*P* < 0.0001) with female age and nest initiation date as the most important predictors in whether or not an individual will lay a second clutch of eggs after successfully rearing a first clutch (Table 1). We found that older prothonotary warbler females are more likely to double brood (*P* < 0.0001). Specifically, compared to first and second year breeders, third and fourth year breeders are two times more likely, and fifth and sixth year breeders are three times more likely to double brood (Fig. 2A). Even when individual females whose age was not known with certainty were removed from the analysis (see methods), age was still a significant predictor of double brooding (*P* < 0.0001). We also found that females that double brooded tended to lay their first clutch earlier (May 1, SD = 5.75 days) compared to females that initiated their first clutch later (May 5, SD = 5.5 days) (Fig. 2B). We found that older females have earlier clutch initiation dates (*P* < 0.0001, Table 2). Because we do not have data on female arrival dates, we were not able to determine if earlier arrivers lay their eggs earlier.

**Table 1:** Output from generalized linear mixed models predicting the probability that a female prothonotary warbler will lay a second clutch of eggs after laying a successful first clutch (i.e., double brood). The top panel is for all individuals and the bottom panel is for only the individuals for which we had consecutive years of data so that we could assess the effect of double brooding in previous years (YRn-1). Clutch size and clutch initiation date are for the first clutch of eggs laid in a season. In both models, female band number and year were entered as random effects to account for the repeated sampling of birds over time

Model Set	Parameter	Estimate	SE	*P*
All data *N* = 2027	Intercept	7.3283	1.9634	<0.001
	Clutch size	0.0104	0.1232	0.933
	Clutch initiation date	−0.0792	0.0131	<0.001
	Female age	0.1862	0.0492	<0.001
Subset *N* = 611	Intercept	5.0926	3.1642	0.1075
	Clutch size	0.1593	0.2103	0.4487
	Clutch initiation date	−0.0632	0.0215	0.0032
	Female age	0.1109	0.0876	0.2054
	Double Brood_(YRn-1)_	0.6614	0.2723	0.0152

**Table 2:** Rate of double brooding and the mean clutch initiation date across female ages in prothonotary warblers. Mean clutch initiation dates are rounded to the nearest day. Females greater than 6 years were combined due to small sample sizes for these older birds

Years Breeding	*N*	Number to double brood	Percentage to double brood	Mean clutch initiation date (±SD)
1	317	27	7.9	May 6 (5.4)
2	560	75	11.8	May 4 (5.5)
3	452	101	18.3	May 3/4 (5.4)
4	199	58	22.6	May 2 (5.3)
5	105	32	23.4	May 3 (5.3)
6+	74	27	26.7	May 3 (5.5)

To assess whether reproductive effort in the previous year affected the probability of double brooding, we looked specifically at individuals for whom we had >1 consecutive year of data and who were at least 2 years old (*N* = 611 events and 398 individuals). We found that birds that double brooded in 1 year were more likely to double brood the following year; 41.1% of females that double brooded in the previous year did so the next whereas only 20.9% of females that did not double brood in the previous year did so the next (Fig. 2C). Nest initiation date was still a predictor of double brooding in this subset of females; however, female age was not (Table 1). Female age is likely no longer a predictor of double brooding in this model because first year breeders are not included in the analysis.

### Proportion of females double brooding each year

**Figure 3:**
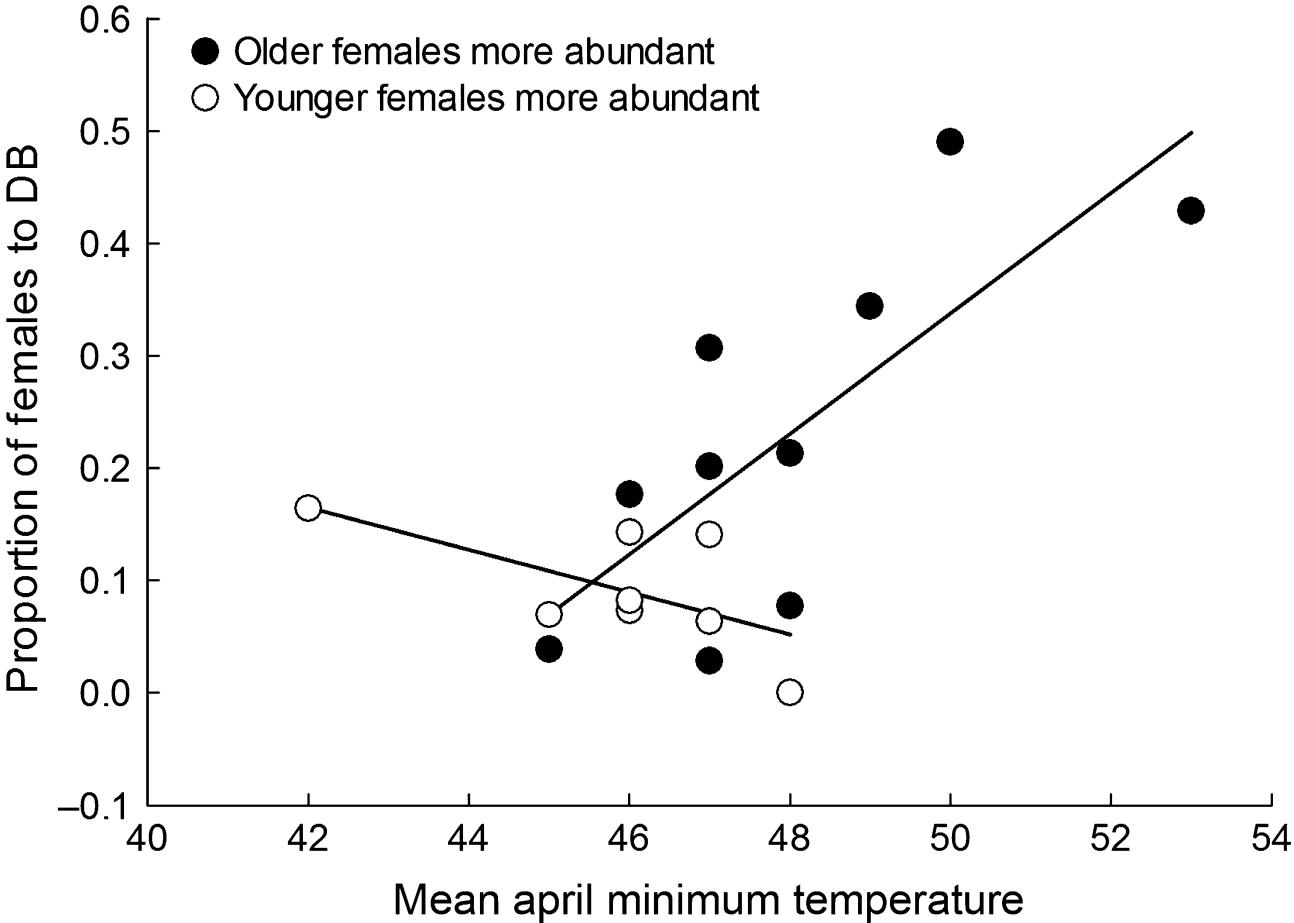
The predicted probability of prothonotary warblers double brooding in a given year depends on mean April minimum temperature, but this relationship varies with female age. During years when older females (at least 3rd year breeders) make up more than half of the breeding population, there is an increase in double brooding with spring temperature (*P* = 0.011, *R*^2^ = 0.57), but this is not the case when younger females (1st and 2nd year breeders) make up more than half of the breeding population (*P* = 0.096, *R*^2^ = 0.39).

We found significant interannual variation in the proportion of birds that double brood and the rate of double brooding seems to be generally increasing in recent years (Table 3, *P* = 0.03, *R*^2^ = 0.26). The lowest rates were in 2004, 2006, and 2007 all with <5% of the females double brooding. The highest rates were in 1998, 1999, and all years since 2008, all with more than 20% of the females double brooding. In the multiple regression model, we found the proportion of females that were 3 years old and older in a given year as well as the interaction between the proportion of older females and April mean minimum temperature to be significant predictors of annual double brooding rate (Table 4). This interaction suggests an age-specific response to early spring temperature whereby older females are more likely and younger females are less likely to double brood when early spring temperatures are warmer (Fig. 3). Mean minimum April temperature is increasing by about 0.23°F/year (*P* = 0.020, *R*^2^ = 0.29) in our study area.

**Table 3:** Summary of double brooding rates in prothonotary warblers in the Lower James River, VA over the 17 years of the study, and the percentage of older females (at least 3 years old)

Year	Number of Birds Breeding	Number Double Brooding	Percentage that Double Brood	Percentage of females ≥3 years
1995	70	6	8.57	32.9
1996	104	7	6.73	37.5
1997	114	21	18.42	38.6
1998	144	35	24.31	68.8
1999	140	30	21.43	57.1
2000	135	22	16.30	36.3
2001	106	10	9.43	48.1
2002	116	9	7.76	53.4
2003	168	24	14.29	45.8
2004	143	0	0.00	46.9
2005	158	13	8.23	44.3
2006	141	4	2.84	51.1
2007	156	6	3.85	58.9
2008	157	54	34.39	67.5
2009	98	42	42.86	64.3
2010	46	13	28.26	59.5
2011	51	25	49.02	62.2
2012	65	20	30.76	55.6

**Table 4:** Output from the multiple regression model assessing annual variation in the proportion of female prothonotary warblers that double brood in a given year along the Lower James River, VA

Parameters	Estimate	SE	*P*
Intercept	−44.82	109.66	0.6900
Mean Minimum April Temp	1.357	1.0597	0.2244
Mean Clutch Initiation Date	−0.2461	0.7907	0.7610
Proportion of older females (3Y+)	0.4735	0.2141	0.0471
Proportion of 3Y+: Mean Minimum April Temp	0.2893	0.0858	0.0056
Proportion of 3Y+: Mean Clutch Initiation Date	−0.1683	0.0930	0.0954

**Figure 4:**
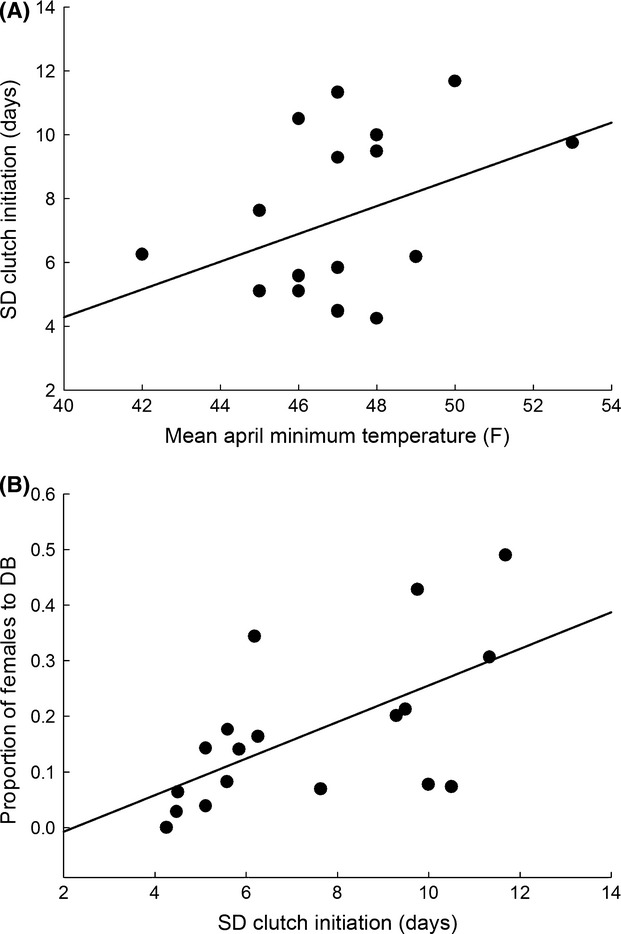
In years with warmer spring temperatures in Virginia, there is slightly more variation in prothonotary warbler clutch initiation date (A, SD = Standard deviation, *P* = 0.11, *R*^2^ = 0.15) and more variation in clutch initiation is correlated with a higher rate of double brooding in this species (B, *P* = 0.008, *R*^2^ = 0.36).

Mean nest initiation date, while a significant predictor of double brooding at the individual level, was not a significant factor in accounting for annual variation in population-level double brooding rate (*P* = 0.7610). Mean nest initiation date varied from 120 (April 30) to 130 (May 10) across years, and did not increase or decrease linearly from 1995 to 2012 (*P* = 0.5974), with increasing spring temperature (*P* = 0.6244), nor with the mean age of breeding females (*P* = 0.4177). We wondered then how much variation there was in nest initiation *within* a season and found that warmer spring temperatures are correlated with greater variation in nest initiation date (*P* = 0.090, Fig. 4A). Furthermore, in years with less synchronous breeding (i.e., increased variation in start date), there was more double brooding in the population (*P* = 0.017, Fig. 4B).

## Discussion

### Probability of individual female double brooding

Our results support what others that have found regarding age and the timing of nest initiation as important predictors of individual double brooding. While evidence of an age effect in our species seems quite strong (Fig. 2A), the effect diminished when first year breeders were excluded (Table 1). Nest initiation, however, remained a strong predictor in both cases which suggests that age may be less important than timing, especially considering that older females tend to initiate nests earlier. Indeed, others have found that age-specific variation in reproduction can be primarily due to early territory establishment by older birds and the subsequent ability to lay more than one clutch in a season (Low et al. 2007). This seems to be the case in our study species though we did not monitor arrival date for females. Other studies show that early nest initiation follows early arrival in both resident (Low et al. 2007) and migratory species (Part 2001; but see Morton and Derrickson 1990).

Our study is one of few demonstrating how the probability of double brooding varies in a population of known-aged individuals and interestingly seems to increase linearly with age with no sign of senescence. Other bird studies demonstrate some degree of senescence with peak reproduction occurring in middle-aged birds (2–4 years) that decreases thereafter (Robertson and Rendell 2001; Low et al. 2007; Bouwhuis et al. 2009), but these studies focus on clutch size or numbers of young fledged per nest and not specifically on the number of broods produced. Double brooding rates that increase linearly with age indicate that it may be a skill that is acquired with age in prothonotary warblers, and once an individual has been successful in such endeavors, they are more likely to repeat it. Our study demonstrates that prior experience seems to play a significant role in whether or not a bird will double brood. Female prothonotary warblers that double brood fledged nearly twice the number of offspring as females who did not, indicating a true reproductive benefit to this behavior. That older females are significantly more likely to double brood suggests that younger individuals are constrained in their reproductive performance either physiologically or in lack of skills (Forslund and Part 1995). However, nearly 8% of first year breeding prothonotary warblers double brooded throughout the 17 years of our study suggesting that this option is indeed possible for young and inexperienced birds, possibly only in “good” years when there are abundant resources and decreased competition (Aroyyo et al. 2007). It is possible that younger females withhold reproductive effort, especially in poor conditions, in an attempt to increase longevity and lifetime reproductive success (Forslund and Part 1995). Ultimately, multiple factors drive age-specific variation in reproduction (Forslund and Part 1995), and our data demonstrate that timing of reproduction and experience in older birds are significant predictors of performance.

### Proportion of females double brooding each year

We found significant variation in the rate of double brooding across years (Table 3). Two factors that contribute significantly to this variation are the proportion of older females (3Y+) in the population and the interaction of the proportion of older females and mean April minimum temperature (Table 4, Fig. 3). The interaction between female age and April temperature may be explained by the fact that younger females arrive later and ostensibly miss an earlier and narrower caterpillar (Lepidoptera larva) peak in warm years, leading to less double brooding in this younger age class. At the same time, older birds arrive earlier and are better able to take advantage of the earlier peak in resources. Caterpillars are the most abundant and available food source for avian insectivores upon arrival to the breeding grounds in early spring (L. Bulluck, pers. obs.) before foliage quality decreases (Jones and Despland 2006). Studies in Europe have found that earlier and narrower peaks in caterpillar abundance have been documented to result from elevated temperatures (Buse et al. 1999; Smith et al. 2011) and to have negative effects on birds that cannot time their reproduction to take advantage of it (Buse et al. 1999; Visser et al. 2003; Husby et al. 2009; Reed et al. 2013). Interestingly, we found that older females responded favorably to warmer temperatures possibly because they are better able to take advantage of narrower and earlier peaks in resources. Our research is the first, to our knowledge, to document how different age classes of birds may respond differently to the timing of resource pulses that may be mediated by arrival timing and nest initiation.

Somewhat surprisingly, mean nest initiation date was not a significant factor in accounting for annual variation in population-level double brooding rate. However, our data suggest that years with warmer spring temperatures have a higher variation in nest initiation date (Fig. 4A) and that variation in nest initiation is correlated with more double brooding (Fig. 4B). This further supports our finding of an age–temperature interaction. In cooler springs, broader and later peaks in caterpillar biomass (Buse et al. 1999; Smith et al. 2011) result in females of all ages (and arrival dates/conditions) laying their eggs more synchronously because all individuals were able to take advantage of this resource. Alternatively in warmer years, there may be less synchrony in nest initiation due to older females being disproportionately able to take advantage of narrower and earlier resource peaks. Schaper et al. (2012) revealed in a controlled aviary setting that the timing of egg laying is genetically controlled in great tits and that increases in temperatures caused the ‘early layers’ to advance laying and the ‘late layers’ to delay laying, which could lead to the same pattern we see with increased variation in nest initiation.

In years with less synchronous breeding (i.e., warmer years with increased variation in start date), there was more double brooding in the population (*P* = 0.017, Fig. 4B). Less synchronous breeding may result in decreased competition for resources during the demanding nestling feeding period. Prothonotary Warblers feed their nestlings both caterpillars and mayflies (*Ephemeroptera*) equally (Petit 1999; L. Bulluck, pers. obs) and mayflies begin to emerge once water temperature increases sufficiently (Chadwick and Feminella 2001), typically in mid-late May when the first clutch of prothonotary warbler nestlings are being fed in our system (L. Bulluck, pers obs). To fully tease apart the mechanisms behind these relationships, caterpillar and mayfly abundance need to be tracked over time for multiple years along with the timing and success of warbler reproduction. Other studies have demonstrated the importance of resource availability in explaining annual variation in reproduction (Rodenhouse and Holmes 1992; Morrison and Bolger 2002; Nagy and Holmes 2004; Husby et al. 2009; Reed et al. 2013), and we expect that variation in resources is likely driving the annual variation in reproduction in our study population as well.

We show that the costs of raising more than one brood per season are not prohibitive as evidenced by the fact that birds do so in consecutive years (Fig. 2C); however, a survival analysis comparing birds that double brood with those that do not will lead to a better understanding of the costs associated with this behavior. We would expect any such costs to be greater for younger individuals, and when environmental conditions are poor (Aroyyo et al. 2007) as would be the case in years with restricted resource pulses. Our study both corroborates prior research with regard to age-specific variation in reproduction while adding valuable new findings. Specifically, ours is one of the few studies of a large number of known-aged individuals that spans nearly two decades, enabling us to demonstrate how populations respond to environmental cues differently depending on the age structure of its members.
